# A Systematic Review and Network Meta-analysis of Empirical Antibiotics for Complicated Urinary Tract Infections

**DOI:** 10.1093/ofid/ofag592

**Published:** 2026-09-26

**Authors:** Lior Cohen-Yatziv, Emily Gilbert, Ruiwen Zhou, Maria Cristina Vazquez Guillamet

**Affiliations:** Division of Infectious Diseases, Department of Medicine, University of Illinois Chicago, Chicago, Illinois, USA; Library of the Health Sciences, University of Illinois Chicago, Chicago, Illinois, USA; Center for Biostatistics and Data Science, Washington University School of Medicine, St. Louis, Missouri, USA; Division of Pulmonary and Critical Care Medicine, Department of Medicine, Washington University in St. Louis School of Medicine, St. Louis, Missouri, USA; Division of Infectious Diseases, Department of Medicine, Washington University in St. Louis School of Medicine, St. Louis, Missouri, USA

**Keywords:** antimicrobial stewardship, beta-lactam antibiotics, complicated urinary tract infections, network meta-analysis, randomized controlled trials

## Abstract

**Background:**

Empiric antibiotic selection for complicated urinary tract infections (cUTIs) is increasingly complex due to the rising prevalence of multidrug-resistant organisms. We conducted a network meta-analysis (NMA) to compare clinical efficacy and safety among existing empiric treatment options.

**Methods:**

We performed a frequentist random-effects NMA of randomized controlled trials evaluating single-agent empiric regimens for adults with cUTIs. The primary endpoints were clinical response (CR), microbiological response, and serious adverse events (AEs). Ceftriaxone served as the common comparator.

**Results:**

Forty-seven trials were identified through a systematic search. In the primary analysis of 17 studies published after the year 2000, evaluating agents currently accessible in the United States, in which baseline ceftriaxone non-susceptibility among Enterobacterales averaged approximately 15%. Ceftriaxone demonstrated CR rates comparable to broader-spectrum alternatives. Although some agents achieved higher microbiological eradication, these differences did not translate into superior clinical cure rates. Safety profiles were stable across antibiotic classes, with no significant differences in serious AEs compared to cephalosporins.

**Conclusions:**

Ceftriaxone remains an effective empiric option for hemodynamically stable adults with cUTIs in settings with comparable resistance prevalence. These findings provide evidence to support antimicrobial stewardship efforts that prioritize narrower-spectrum agents, preserving broader-spectrum therapies for patients at highest risk for resistant pathogens.

## Teaser

In this network meta-analysis for cUTI, ceftriaxone achieved clinical cure rates comparable to broader-spectrum alternatives, with no difference in serious adverse events. These findings support narrower empiric therapy with ceftriaxone for hemodynamically stable adults in settings with similar resistance prevalence.

Urinary tract infections (UTIs) are a major cause of morbidity, hospitalization, and antimicrobial utilization worldwide [1]. They are associated with significant morbidity and mortality, often leading to long-term sequelae. Mortality rates have been reported to be as high as 15% among hospitalized patients with complicated UTIs (cUTIs) [2]. These infections cause a major burden on healthcare systems worldwide, with annual costs estimated to reach $44 billion [3]. Given their clinical impact and broad heterogeneity, cUTIs require timely and effective empiric therapy, yet the optimal initial antibiotic choice remains uncertain.

The 2025 IDSA cUTI guideline introduced a classification that focuses on the presence or absence of localized or systemic symptoms, particularly fever or other features suggesting that infection has progressed beyond the bladder, together with factors readily apparent at the point of care such as vital signs and the presence of an indwelling urinary catheter [4]. cUTIs therefore span a broad clinical spectrum, including pyelonephritis, UTI accompanied by systemic signs of infection, and UTI in the setting of structural or functional urinary tract abnormalities or indwelling catheters. While cUTI and uncomplicated UTI are caused by a largely overlapping microbiological spectrum, the clinical phenotype of cUTI, characterized by systemic inflammatory signs and an elevated risk of progression to urosepsis, has historically warranted broader empiric antimicrobial coverage and longer treatment durations [5].

Most cUTIs are caused by gram-negative bacilli (GNB), predominantly Enterobacterales [6]. The increasing global prevalence of antibiotic-resistant organisms (AROs), including extended-spectrum β-lactamase (ESBL)-producing Enterobacterales and carbapenem-resistant GNB, has complicated empiric management and driven the development of several novel β-lactam/β-lactamase inhibitor combinations [7, 8]. These trends increase pressure toward early use of broad-spectrum antibiotics [9].

Existing randomized trials evaluating empiric therapy for cUTI are characterized by heterogeneous patient populations and variability in the choice of comparators. This variability limits the interpretability of individual studies to inform empiric treatment selection.

In clinical practice, therapy is often initiated empirically, before culture results are available, and many patients lack recent microbiologic data or have uncertain prior antimicrobial exposure. The increasing prevalence of AROs limits safe empiric options and raises the risk of discordant coverage. Broad-spectrum antibiotics, including carbapenems and piperacillin–tazobactam, are frequently used empirically despite limited indications for many hospitalized patients with cUTI and other infectious syndromes [10–12]. Unnecessary early broad-spectrum use contributes to antimicrobial resistance, exposes patients to avoidable adverse events (AEs), and has not consistently demonstrated improved outcomes when susceptible pathogens are present [13–16]. Moreover, prior studies have shown that when pathogens are susceptible, clinical outcomes achieved with narrower β-lactams, such as third-generation cephalosporins, are comparable to those achieved with broader agents [17]. These observations reinforce the importance of identifying narrower agents that remain effective for empiric therapy in hemodynamically stable patients. The IDSA 2025 cUTI guideline operationalizes empiric antibiotic selection through a step-wise approach that stratifies patients by severity of illness, then considers patient-specific risk factors for resistant uropathogens, patient-specific considerations such as allergies and drug–drug interactions, and, in septic patients, the local antibiogram. For patients with suspected cUTI without sepsis, the guideline conditionally recommends initially selecting among third- or fourth-generation cephalosporins, piperacillin–tazobactam, or fluoroquinolones, rather than carbapenems, newer β-lactam/β-lactamase inhibitor combinations, cefiderocol, plazomicin, or aminoglycosides [4]. The 2024 UTI WikiGuidelines consensus statement complements this framework by highlighting that high-quality prospective data on empiric antibiotic selection for cUTI remain limited [18]. These observations underscore the value of synthesizing the available randomized evidence.

Network meta-analysis (NMA) allows integration of direct and indirect evidence from multiple randomized trials, enabling simultaneous comparison and ranking of several antibiotics even when head-to-head trials are lacking [19]. This method has been successfully applied in many infectious syndromes [20, 21].

Given the rising prevalence of AROs and the necessity of initiating therapy before pathogen identification, there is a need for a comparative hierarchy of existing treatments. To address this, we conducted a frequentist random-effect NMA to simultaneously evaluate the clinical efficacy, microbiological eradication, and safety profiles of current single-agent regimens for the empiric management of cUTI.

## METHODS

This systematic review was performed in accordance with the Preferred Reporting Items for Systematic Reviews and Meta-Analysis (PRISMA) statement 2020 [22].

### Search Strategy

The search strategy was written by a medical librarian, combining controlled vocabulary terms and keywords. Terms for complicated urinary tract infection, hospital-acquired urinary tract infection, and pyelonephritis were combined with terms for antibiotics, the general concept, and individual drug names. Validated search hedges were applied to filter for randomized controlled trials (RCTs) and filter out pediatric populations, and the publication date was filtered for 1990 to present. The entire search strategy and terms are provided in (Supplementary 1). Searches were completed in PubMed (MEDLINE), EMBASE, Scopus, and Cochrane CENTRAL, yielding 5739 citations. Three retractions and 1408 duplicates were removed using EndNote, and the remaining 4328 studies were uploaded to COVIDence [23, 24].

We included RCTs evaluating at least 2 empiric systemic antibiotic regimens, defined as therapy initiated prior to pathogen identification in adults (≥18 years) hospitalized with cUTI or pyelonephritis. Fixed-dose β-lactam/β-lactamase inhibitor combinations and other single-formulation co-formulated regimens were considered single agents and were eligible for inclusion. We excluded trials involving uncomplicated UTIs, outpatient populations, pediatric patients, concurrent multi-antibiotic empiric regimens (defined as 2 or more systemically administered antibiotics used simultaneously as initial therapy), and studies that directed therapy toward known AROs or utilized susceptibility results to guide initial agent selection. When studies included mixed infection types (eg, intra-abdominal, respiratory), only data specific to the cUTI subgroup were extracted, provided they included more than 10 patients in each comparison group.

### Data Extraction and Variables of Interest

For each eligible study that met our criteria, we extracted data using a standardized form. The study was reviewed in detail, and information about clinical efficacy, microbiological efficacy, and AEs was collected. Emphasis was placed on the intention-to-treat population when reported. Clinical response (CR) was determined by the authors of the individual studies and reflected a CR at a prespecified test-of-cure visit or transition from intravenous (IV)-to-oral therapy. Similarly, microbiological response (MR) was determined as microbiological eradication at a prespecified test-of-cure visit or other pre-defined date. When this was examined on multiple dates—we chose the earliest date available.

For AEs, we focused on AEs that were deemed serious and were defined according to each trial's prespecified safety definitions. Although individual studies did not uniformly reference a single regulatory standard, reported serious AEs were broadly consistent with International Council for Harmonisation Good Clinical Practice criteria [25].

Separate nodes in the network were generated for each unique antibiotic, and no duplicates were made due to differences in duration of therapy, medication dose, duration of infusion, or frequency of administration.

### Risk of Bias and Certainty of Evidence Assessment

Risk of bias was assessed for each included study using the Cochrane Risk of Bias 2 (RoB 2) tool [26]. The overall certainty of evidence for the primary outcomes (CR and MR) was evaluated using the GRADE (Grading of Recommendations Assessment, Development, and Evaluation) framework specifically adapted for NMA [27]. Following a standardized 4-step approach, we assessed the network for study limitations (risk of bias), indirectness, inconsistency, imprecision, and publication bias. The certainty was categorized as high, moderate, low, or very low. Full methodological details and individual study assessments are provided (Supplementary 2).

### Data Synthesis and Analysis

A frequentist random-effects NMA was performed using the *netmeta* package in R (version 4.4.3), employing restricted maximum likelihood (REML) estimation to model between-study variance [19]. This model assumes that the true effect of each treatment is drawn from a distribution due to the heterogeneity in the study populations and designs. Ceftriaxone was utilized as the common comparator since it is widely available, among the most commonly chosen empiric agents for hospitalized cUTI in settings where local susceptibility supports third-generation cephalosporin coverage, and offers favorable pharmacokinetic properties, high urinary concentrations, and proven efficacy for Enterobacterales infections, while avoiding unnecessary antipseudomonal, ESBL, or anti-anaerobic coverage [28, 29].

We visualized relative treatment effects from the random-effects NMA as forest plots showing odds ratios with 95% confidence intervals for each regimen versus ceftriaxone, separately for clinical and microbiologic response. Estimates were generated using inverse-variance weighting with between-study heterogeneity (τ^2^) derived from the REML-based consistency model. Heterogeneity was assessed using Cochran's Q statistic, tau^2^, and the I^2^ statistic across each network. All analyses adhered to PRISMA-NMA reporting standards [30].

We determined that the enrolled cUTI populations, empiric treatment settings, and outcome definitions across trials were sufficiently similar to allow for indirect comparisons.

Consistency was evaluated using both global and local approaches. Globally, the design-by-treatment interaction model tested for disagreement between direct and indirect evidence across the full network. Locally, node-splitting was applied to all treatment comparisons with available direct and indirect data, and no significant inconsistency was detected. For class-level adverse event analyses, we combined agents within antibiotic classes and evaluated within- and between-design Q components to assess coherence of evidence. After completing the initial analysis and NMA, we conducted several subset analyses (Supplementary 3). We chose to focus on studies beginning from the year 2000 and included only those assessing drugs that are easily accessible in the United States.

### Patient Consent Statement

This study is a systematic review and NMA of previously published, aggregate-level data, no patient consent and ethical approval were required.

## RESULTS

### Trial Selection

After deduplication and automated screening, 1951 records underwent title and abstract screening and 201 full-text review from which 47 studies met inclusion criteria (Figure 1).

**Figure 1.**
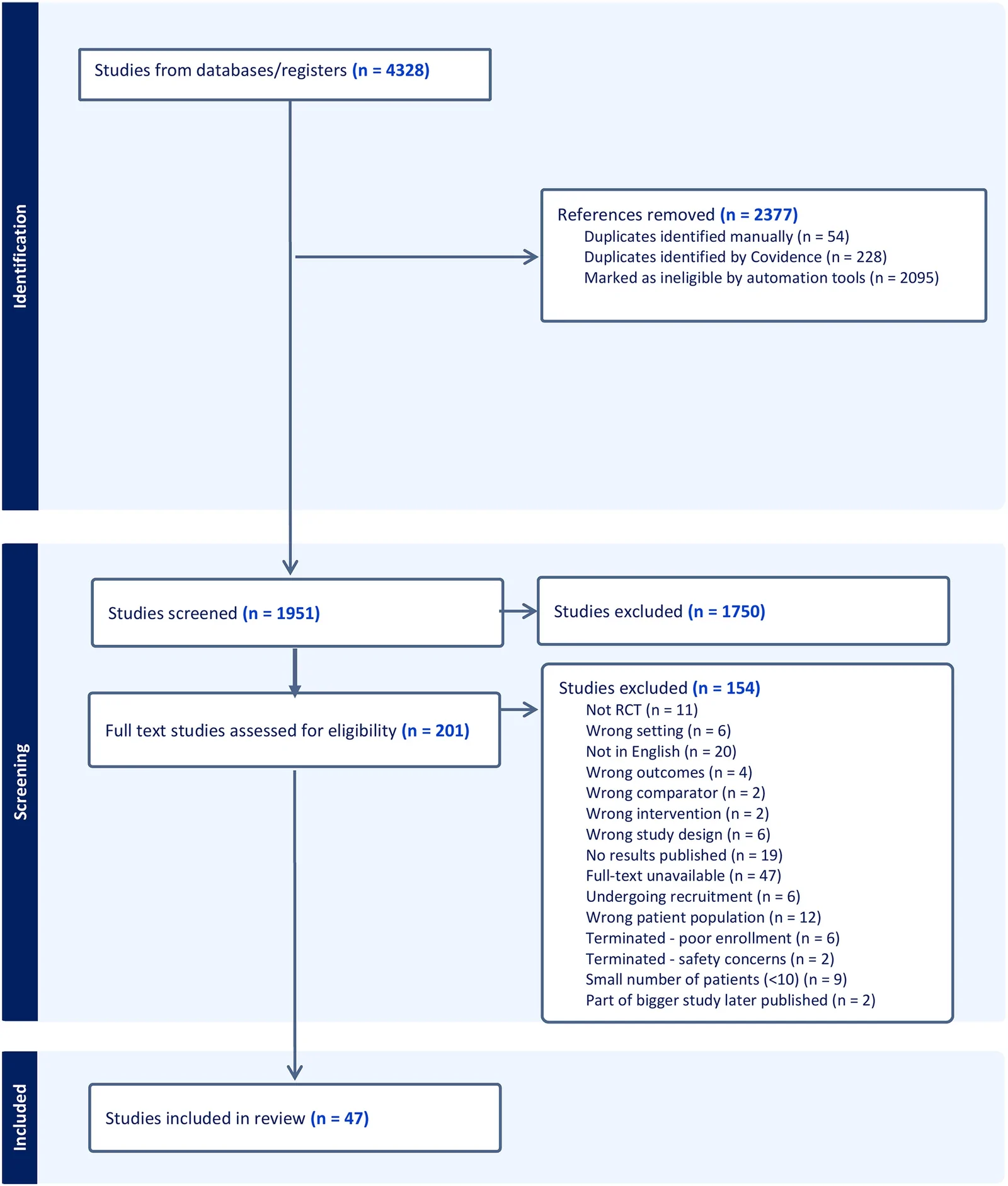
PRISMA flow diagram of study identification and selection.

Clinical response and MR were assessed across all planned sub-analyses; Table 1 summarizes only studies published from 2000 onward that evaluated antibiotics currently available in the United States.

**Table 1.** Titles and Information of the Randomized Controlled Trials Published From 2000 Evaluating Empiric Antibiotic Regimens for Complicated Urinary Tract Infections in Adults, Restricted to Drugs Currently Available in the United States

Title	Authors	Published Year	Journal	DOI
Sulopenem for the treatment of complicated urinary tract infections including pyelonephritis: a phase 3, randomized trial	Dunne, M. W.; Aronin, S. I.; Das, A. F.; Akinapelli, K.; Breen, J.; Zelasky, M. T.; Puttagunta, S.	2023	Clin Infect Dis	10.1093/cid/ciac704
Oral tebipenem pivoxil hydrobromide in complicated urinary tract infection	Eckburg, P. B.; Muir, L.; Critchley, I. A.; Walpole, S.; Kwak, H.; Phelan, A. M.; Moore, G.; Jain, A.; Keutzer, T.; Dane, A.; et al.,	2022	N Engl J Med	10.1056/NEJMoa2105462
Levofloxacin versus ceftriaxone for the treatment of acute pyelonephritis in Iranian adults	Tehrani, S.; Elyasi, F.; Abolghasemi, S.	2021	Infect Disord Drug Targets	10.2174/1871526520999200727154214
Fosfomycin for injection (ZTI-01) versus piperacillin-tazobactam for the treatment of complicated urinary tract infection including acute pyelonephritis: ZEUS, a phase 2/3 randomized trial	Kaye, K. S.; Rice, L. B.; Dane, A. L.; Stus, V.; Sagan, O.; Fedosiuk, E.; Das, A. F.; Skarinsky, D.; Eckburg, P. B.; Ellis-Grosse, E. J.	2019	Clin Infect Dis	10.1093/cid/ciz181
Effect of meropenem-vaborbactam vs piperacillin-tazobactam on clinical cure or improvement and microbial eradication in complicated urinary tract infection: the TANGO I randomized clinical trial	Kaye, K. S.; Bhowmick, T.; Metallidis, S.; Bleasdale, S. C.; Sagan, O. S.; Stus, V.; Vazquez, J.; Zaitsev, V.; Bidair, M.; Chorvat, E.; Dragoescu, P. O.; Fedosiuk, E.; Horcajada, J. P.; Murta, C.; Sarychev, Y.; Stoev, V.; Morgan, E.; Fusaro, K.; Griffith, D.; Lomovskaya, O.; Alexander, E. L.; Loutit, J.; Dudley, M. N.; Giamarellos-Bourboulis, E. J.	2018	JAMA	10.1001/jama.2018.0438
Cefiderocol versus imipenem-cilastatin for the treatment of complicated urinary tract infections caused by Gram-negative uropathogens: a phase 2, randomised, double-blind, non-inferiority trial.	Portsmouth S; van Veenhuyzen D; Echols R; Machida M; Ferreira JCA; Ariyasu M; Tenke P; Nagata TD	2018	Lancet Infect Dis	10.1016/S1473-3099(18)30554-1
Prospective, randomized, double-blind, Phase 2 dose-ranging study comparing efficacy and safety of imipenem/cilastatin plus relebactam with imipenem/cilastatin alone in patients with complicated urinary tract infections	Sims, M.; Mariyanovski, V.; McLeroth, P.; Akers, W.; Lee, Y. C.; Brown, M. L.; Du, J.; Pedley, A.; Kartsonis, N. A.; Paschke, A.	2017	J Antimicrob Chemother	10.1093/jac/dkx139
Ceftazidime-avibactam versus doripenem for the treatment of complicated urinary tract infections, including acute pyelonephritis: RECAPTURE, a phase 3 randomized trial program	Wagenlehner, F. M.; Sobel, J. D.; Newell, P.; Armstrong, J.; Huang, X.; Stone, G. G.; Yates, K.; Gasink, L. B.	2016	Clin Infect Dis	10.1093/cid/ciw378
Ceftolozane-tazobactam compared with levofloxacin in the treatment of complicated urinary-tract infections, including pyelonephritis: a randomised, double-blind, phase 3 trial (ASPECT-cUTI)	Wagenlehner, F. M.; Umeh, O.; Steenbergen, J.; Yuan, G.; Darouiche, R. O.	2015	Lancet	10.1016/s0140-6736(14)62220-0
Comparison of ertapenem and ceftriaxone therapy for acute pyelonephritis and other complicated urinary tract infections in Korean adults: a randomized, double-blind, multicenter trial	Park, D. W.; Peck, K. R.; Chung, M. H.; Lee, J. S.; Park, Y. S.; Kim, H. Y.; Lee, M. S.; Kim, J. Y.; Yeom, J. S.; Kim, M. J.	2012	J Korean Med Sci	10.3346/jkms.2012.27.5.476
Efficacy and safety of ceftazidime-avibactam versus imipenem-cilastatin in the treatment of complicated urinary tract infections, including acute pyelonephritis, in hospitalized adults: results of a prospective, investigator-blinded, randomized study	Vazquez, J. A.; González Patzán, L. D.; Stricklin, D.; Duttaroy, D. D.; Kreidly, Z.; Lipka, J.; Sable, C.	2012	Curr Med Res Opin	10.1185/03007995.2012.748653
Comparing ceftriaxone and cefazolin for treatment of adult acute pyelonephritis; a clinical trial	Ebrahimzadeh, A.; Saadatjoo, S. A.; Tabrizi, A. A.	2010	Iran J Clin Infect Dis	
Intravenous doripenem at 500 milligrams versus levofloxacin at 250 milligrams, with an option to switch to oral therapy, for treatment of complicated lower urinary tract infection and pyelonephritis	Naber, K. G.; Llorens, L.; Kaniga, K.; Kotey, P.; Hedrich, D.; Redman, R.	2009	Antimicrob Agents Chemother	10.1128/aac.00837-08
A double-blind, randomized comparison of levofloxacin 750 mg once-daily for five days with ciprofloxacin 400/500 mg twice-daily for 10 days for the treatment of complicated urinary tract infections and acute pyelonephritis	Peterson, J.; Kaul, S.; Khashab, M.; Fisher, A. C.; Kahn, J. B.	2008	Urology	10.1016/j.urology.2007.09.002
A prospective, multicenter, randomized, double-blind study comparing ertapenem and ceftriaxone followed by appropriate oral therapy for complicated urinary tract infections in adults	Jimenez-Cruz, F.; Jasovich, A.; Cajigas, J.; Jiang, Q.; Imbeault, D.; Woods, G. L.; Gesser, R. M.	2002	Urology	10.1016/s0090-4295(02)01664-3
Piperacillin 2 g/tazobactam 0.5 g is as effective as imipenem 0.5 g/cilastatin 0.5 g for the treatment of acute uncomplicated pyelonephritis and complicated urinary tract infections	Naber, K. G.; Savov, O.; Salmen, H. C.	2002	Int J Antimicrob Agents	10.1016/s0924-8579(01)00481-2
Ertapenem versus ceftriaxone followed by appropriate oral therapy for treatment of complicated urinary tract infections in adults: Results of a prospective, randomized, double-blind multicenter study	Tomera, K. M.; Burdmann, E. A.; Pamo Reyna, O. G.; Jiang, Q.; Wimmer, W. M.; Woods, G. L.; Gesser, R. M.; Albertson, E. A.; Al-Ibrahim, M. S.; Burke, T. F.; Childs, S.; Cox, C.; Eng, R. H. K.; Frank, E.; Gainer Ii, R. B.; Fuenzalida, F. G.; Gezon, J. A.; Herman, T.; Jacoby, K.; Krinsky, A. H.; Landau, R. E.; Moseley, W.; Rangel-Frausto, M. S.; Recinos-Mijangos, G.; Reinhardt, J. F.; Sarshik, S.; Schilling, A.; Tuttle Jr, J.; Walzer, Y.; Wells, G.	2002	Antimicrob Agents Chemother	10.1128/AAC.46.9.2895-2900.2002

DOI, digital object identifier.

Clinical response results are provided for all 47 included studies (network, Supplementary Figure 1; forest plot, Supplementary Figure 2), for studies published from 2000 onward (network, Supplementary Figure 3; forest plot, Supplementary Figure 4), and for studies restricted to agents currently available in the United States (network, Supplementary Figure 5; forest plot, Supplementary Figure 6).

Of the 17 trials, 59% were judged to have a low risk of bias, with concerns in older studies primarily related to open-label designs (Supplementary 2). Following the GRADE-NMA framework, the certainty of evidence for our primary outcomes (CR and MR) averaged at Moderate-High (Supplementary 2). Downgrading occurred primarily due to imprecision in comparisons, where the total number of events was relatively small, or due to risk of bias in open-label trials.

### Network Characteristics

The network of different antibiotic treatments for CR comprised 16 nodes and 17 studies, involving a total of 6997 patients. Treatments were linked through ceftriaxone as a shared comparator, forming a connected network of regimens suitable for indirect comparison (Figure 2).

**Figure 2.**
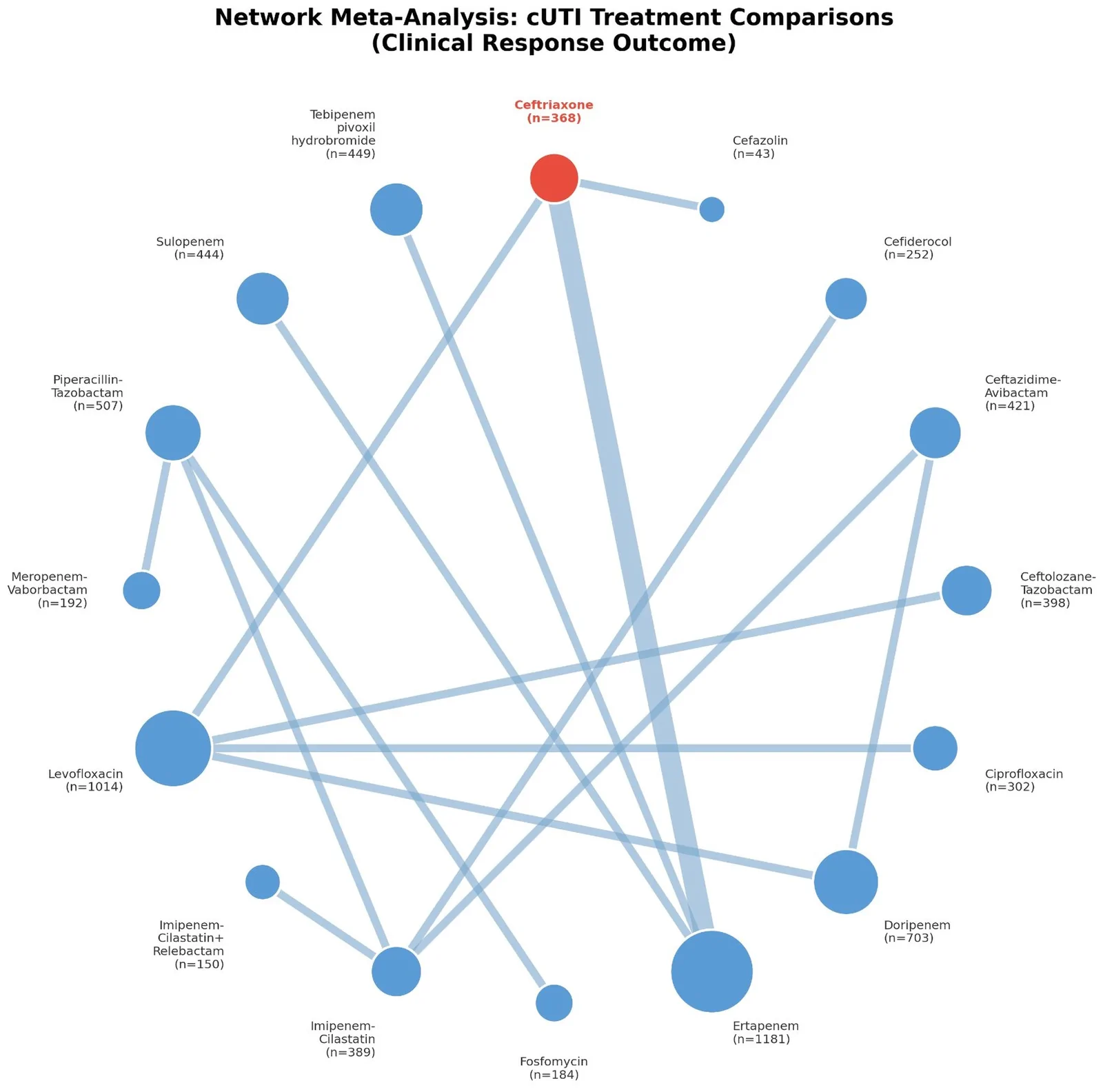
Network plot for clinical response. Each node represents an antibiotic treatment; node size is proportional to the total number of patients in that treatment arm. Edge thickness reflects the number of direct comparisons between treatments. Ceftriaxone serves as the common reference comparator.

The forest plot summarizes the comparative effects of each antimicrobial agent against ceftriaxone for CR, displaying pooled odds ratios with corresponding 95% confidence intervals from the random-effects model. Effect estimates for most agents clustered around the null with overlapping confidence intervals (Figure 3).

**Figure 3.**
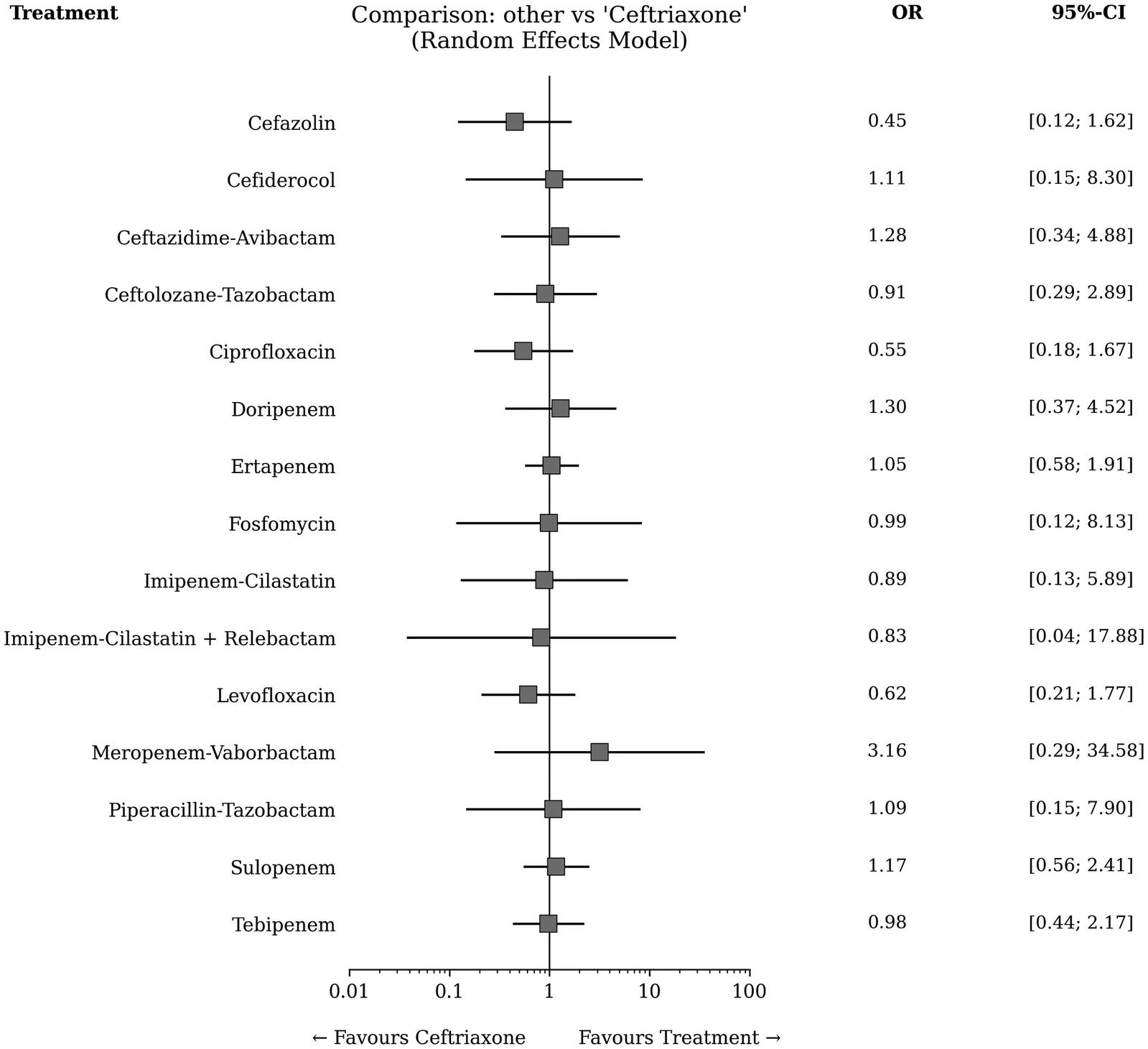
Forest plot for clinical response. Odds ratios (ORs) and 95% confidence intervals (CIs) comparing each treatment to ceftriaxone (reference, OR = 1.00) from the random-effects network meta-analysis. Values greater than 1 favor the treatment; values less than 1 favor ceftriaxone.

In the assessment of between-study heterogeneity, the Cochran Q statistic was 2.816 with 5 degrees of freedom (*P* = .728), indicating no evidence of meaningful heterogeneity across the included studies. The estimated between-study variance was tau^2^ = 0, with a corresponding tau of 0, supporting the absence of dispersion in true effect sizes. The I^2^ statistic was 0%, further confirming that the observed variation was fully compatible with sampling error.

The network of different antibiotic treatments for MR consisted of 16 nodes and 17 studies, containing a total of 7331 patients. Treatments were linked through ceftriaxone as a common comparator, forming a connected network of empiric single-agent regimens that allowed indirect comparison across studies (Supplementary 4, Supplementary Figure 7). Effect estimates varied across agents, with wide confidence intervals reflecting heterogeneity and limited data for some comparisons. (Figure 4). The forest plot for MR summarizes the comparative effects of each antimicrobial agent against ceftriaxone for MR, displaying pooled odds ratios with corresponding 95% confidence intervals from the random-effects model. The Cochran Q statistic was 2.433 with 5 degrees of freedom (*P* = .787), indicating that the observed variation in effect sizes was fully compatible with chance. The estimated between-study variance was tau^2^ = 0, with a corresponding tau of 0, demonstrating no measurable dispersion in true effects. The I^2^ statistic was 0%, confirming the absence of heterogeneity.

**Figure 4.**
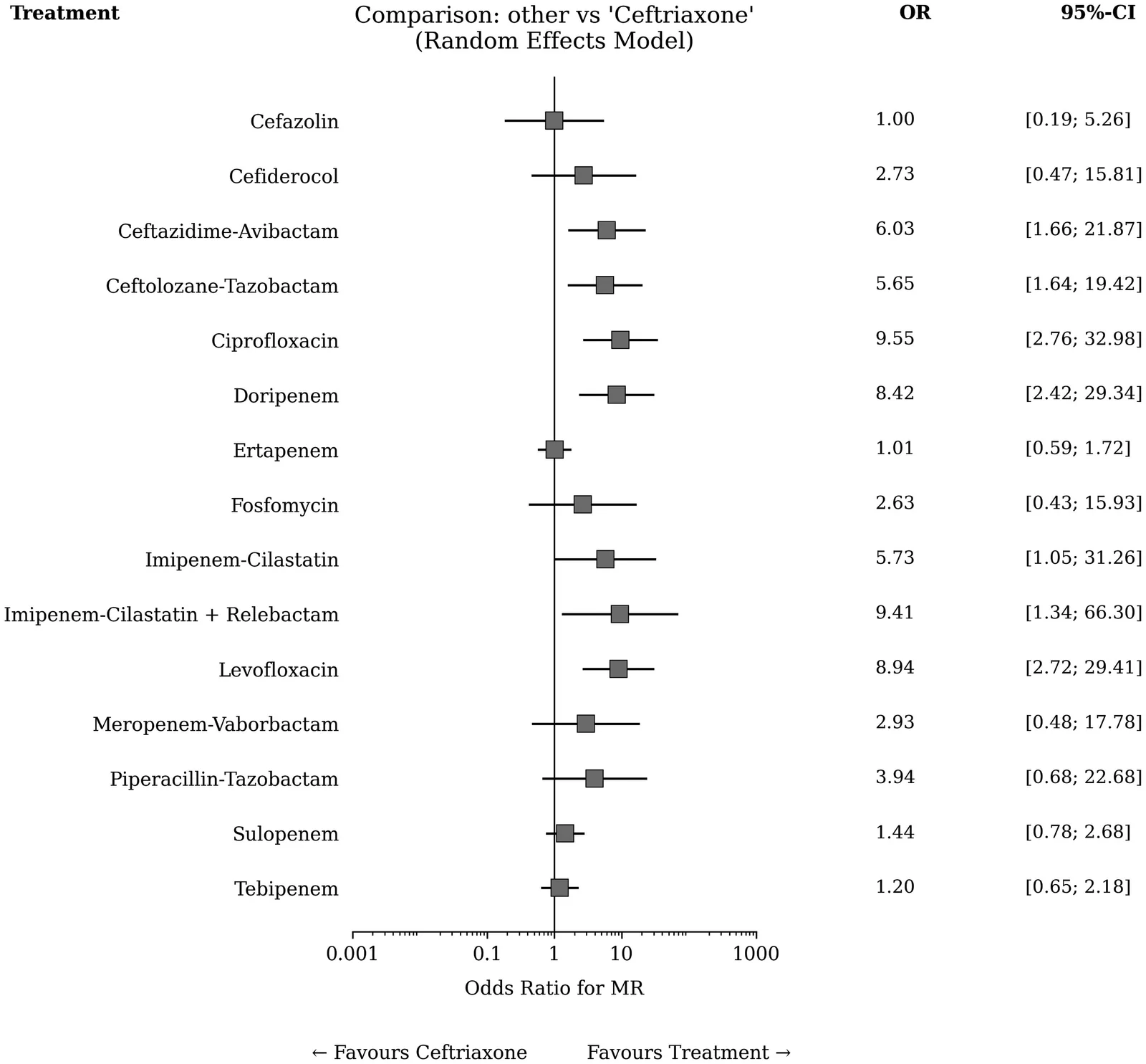
Forest plot for microbiological response. Odds ratios (ORs) and 95% confidence intervals (CIs) comparing each treatment to ceftriaxone (reference, OR = 1.00). Values greater than 1 favor the treatment; values less than 1 favor ceftriaxone.

For AEs, due to sparse reporting of serious AEs across individual trials, multiple treatments were combined within each antibiotic class to permit analysis. This class-level aggregation yielded a well-connected network with good homogeneity and no evidence of inconsistency (Figure 5). In the corresponding forest plot, no antibiotic class demonstrated higher rates of serious AEs compared with cephalosporins, which served as the reference category (Figure 6). Formal assessment of heterogeneity and consistency supported these findings, with a nonsignificant overall Q statistic (Q = 13.23, df = 8, *P* = .10), as well as nonsignificant within-design (Q = 9.88, df = 6, *P* = .13) and between-design components (Q = 3.34, df = 2, *P* = .19).

**Figure 5.**
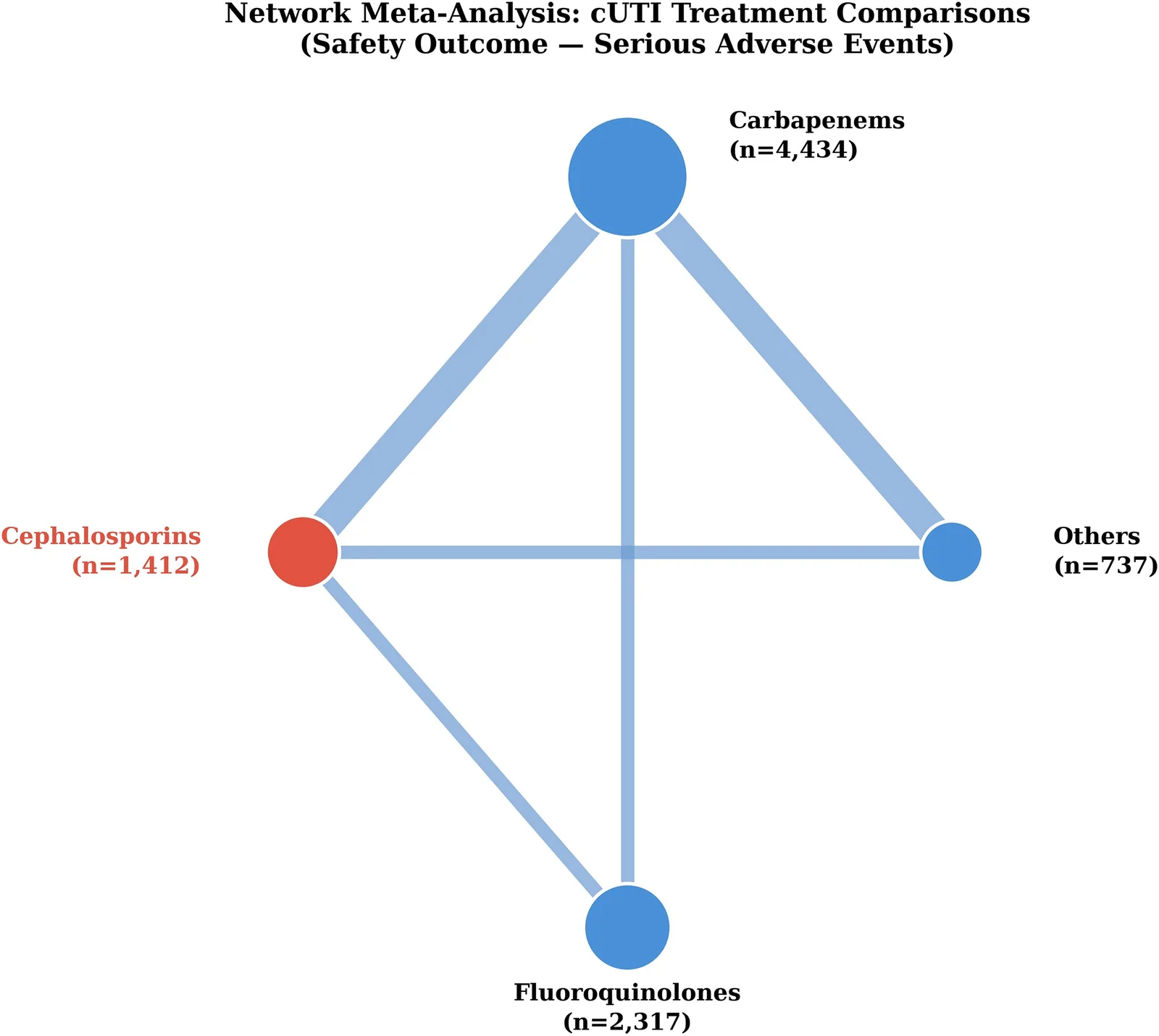
Network plot for serious adverse events analyzed by antibiotic class. Node size reflects total patients; edge thickness reflects the number of inter-class comparisons. Cephalosporins serve as the reference comparator.

**Figure 6.**
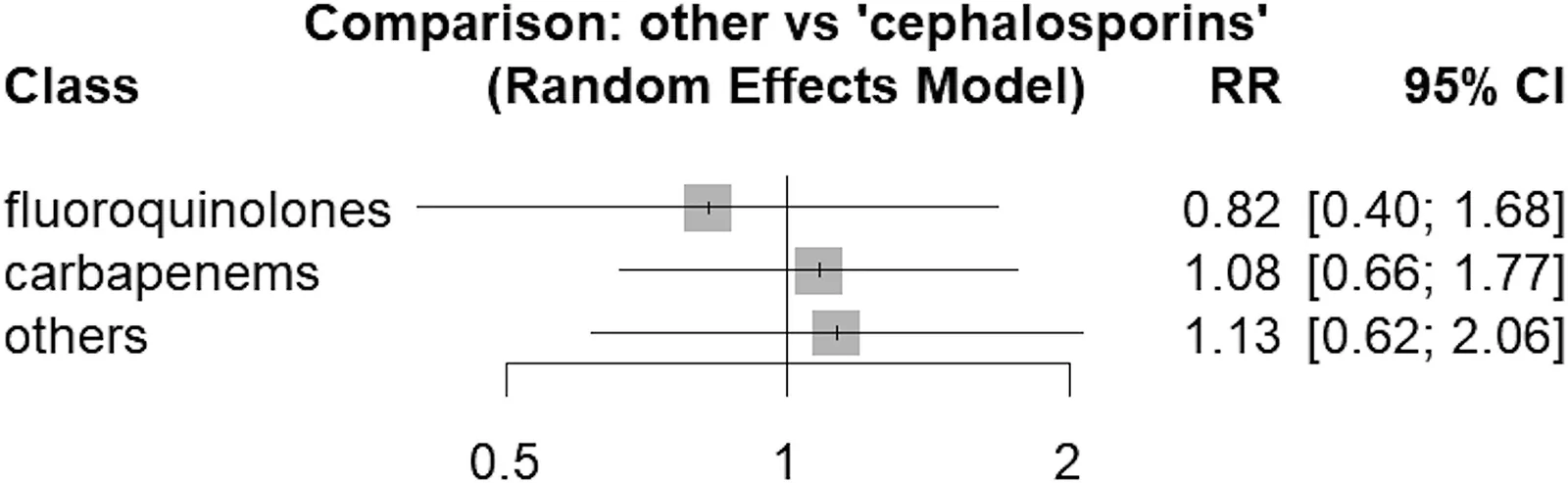
Forest plot for serious adverse events by antibiotic class. Risk ratios and 95% confidence intervals comparing each class to cephalosporins (reference, RR = 1.00).

## DISCUSSION

Our NMA found that ceftriaxone achieved CR rates comparable to broader-spectrum alternatives, demonstrated a similar adverse event profile, and remained a reliable empiric option for cUTI.

Although certain therapeutic classes, most notably novel β-lactam/β-lactamase inhibitor combinations and carbapenems, demonstrated statistically superior MR rates, the clinical utility of these differences is unclear. This microbiological advantage was predominantly observed against Enterobacterales, with a less definitive impact on *Pseudomonas* species. Several non-pharmacologic factors may also contribute: many ceftriaxone-anchored regimens were transitioned to oral β-lactams with limited evidence for short-course dosing, whereas several newer BL/BLI trials maintained all-IV therapy throughout, and total treatment durations varied across trials [31]. This effect peaked post-IV therapy and was attenuated by the final test-of-cure. Many microbiological “failures” likely reflected asymptomatic bacteriuria, rather than a clinical relapse, as these patients remained symptom-free despite persistent or recurrent bacteriuria at the test-of-cure visit. Current guidelines advise against obtaining post-treatment urine cultures in patients who have improved clinically and prioritize symptom resolution. MR therefore remains an imperfect outcome in clinical trials [32, 33].

The clinical management of cUTI follows a biphasic paradigm, transitioning from empiric therapy, initiated during the critical 48-to-72-hour window of clinical uncertainty, to pathogen-directed therapy once antimicrobial susceptibilities are available. Our NMA highlights that despite the introduction of newer beta-lactam/beta-lactamase inhibitor combinations and carbapenems, traditional agents such as ceftriaxone remain a reasonably effective empiric option in non-ARO-endemic areas. As a robust bridge to directed therapy, ceftriaxone achieves comparable clinical success while minimizing unnecessary broad-spectrum usage.

Our findings align with the IDSA 2025 cUTI guideline's risk-stratified empiric approach and antimicrobial stewardship principles that emphasize prioritizing a narrow effective empiric agent [4]. Reserving carbapenems and other broad-spectrum therapies for patients with known or high-risk ESBL-producing pathogens, or patients with sepsis, helps maintain efficacy while limiting collateral damage, selective pressure, and unnecessary AEs [34, 35]. By demonstrating comparable clinical outcomes between ceftriaxone and broader-spectrum alternatives, our NMA quantitatively supports this approach.

The clinical application of these stewardship principles is fundamentally contingent upon local resistance, which exhibits significant geographic heterogeneity. While global surveillance highlights a concerning rise in ESBL-producing *Enterobacterales*, these trends have not yet rendered third-generation cephalosporins obsolete in most clinical settings. In the United States, the prevalence of ESBL-producing isolates remains near 15%, a threshold that closely mirrors the resistance rates reported in the randomized trials analyzed in our NMA, though regional surveillance within the United States varies substantially [36]. Crucially, despite rising concern for resistance, most hospitalized patients with cUTI who are hemodynamically stable do not harbor highly resistant pathogens [35]. This congruence underscores our findings and supports a risk-stratified empiric strategy that preserves broad-spectrum agents for the critically ill while utilizing ceftriaxone as a reliable backbone for the majority of stable patients.

Complementing these efficacy data, our class-level aggregation of AEs demonstrated stable and coherent safety profiles across antibiotic classes, with no class showing higher rates of serious AEs than cephalosporins. While class-level aggregation may mask drug-specific variability, it provides a pragmatic framework for evaluating comparative tolerability in empiric therapy decisions, particularly when individual-agent data are limited. These results suggest that, from a tolerability standpoint, commonly used empiric options are broadly similar, allowing clinical efficacy and stewardship considerations to drive decision-making without the confounding concern of disproportionate toxicity.

Our findings differ from prior NMA, such as that by Wagenlehner et al., which included combination regimens and agents targeting known AROs [37]. They found that novel β-lactam/β-lactamase inhibitor combinations were superior in microbiological eradication compared to older agents. In contrast, our study focused exclusively on single-agent empiric regimens used before pathogen identification, reflecting real-world decision-making for most hospitalized patients. We selected ceftriaxone as the reference comparator to align with stewardship priorities, whereas prior analyses used carbapenems for comparison.

This study has several limitations. As with all meta-analyses, patient-level data were not available, and many trials excluded high-risk groups with bacteremia, obstruction, or immunocompromising conditions; notably, septic shock was identified only in 24 patients. This focus on hemodynamically stable patients aligns with IDSA guidelines, which distinguish treatment strategies according to the patient's clinical severity presentation [4].

We excluded non-English published trials. While this approach may introduce a degree of language bias, prior evidence suggests that this exclusion in conventional meta-analyses rarely alters the direction or significance of treatment effects [38].

Baseline resistance prevalence differed across the constituent trials but, on average, ceftriaxone non-susceptibility among Enterobacterales was near 15%, which limits the generalizability of our pooled estimates to settings whose local resistance epidemiology differs from that of the included studies [36]. Definitions of clinical and microbiologic response also varied. Generally, we judged this acceptable for indirect comparison because outcome ascertainment was anchored on a prespecified test-of-cure assessment combining clinical improvement and microbiologic eradication. We could not account for differences in dosing, IV-to-oral transitions, treatment duration, or evolving clinical breakpoints. Notably, safety outcomes were inconsistently reported. We excluded combination regimens and ARO-targeted therapies, making our findings inapplicable when ESBL or Pseudomonas infection is suspected.

Despite these limitations, our analysis provides clinically relevant evidence supporting ceftriaxone as an effective empiric option for complicated UTIs in hemodynamically stable adults. By demonstrating comparable clinical outcomes to broader-spectrum agents, these findings strengthen the case for a stewardship-aligned approach that preserves broad-spectrum antibiotics for patients most likely to benefit. Future work should prioritize direct comparisons of ceftriaxone with novel agents and the development of more accurate tools to identify patients at elevated risk for AROs. Enhanced risk stratification would allow clinicians to more precisely target broad-spectrum agents to those who truly need them, while safely preserving narrower options for most patients.

## CONCLUSION

In this systematic review and NMA, ceftriaxone demonstrated clinical effectiveness comparable to other agents for the empiric treatment of complicated UTIs, with no meaningful differences in serious AEs across antibiotic classes. Although other regimens showed higher microbiologic eradication rates, these differences did not translate into improved clinical outcomes and are unlikely to alter empiric decision-making. These findings support the use of ceftriaxone as a reliable empiric option for hemodynamically stable adults with no ARO risk factors and not in ARO-endemic areas, reinforcing stewardship efforts to limit unnecessary early exposure to broad-spectrum antibiotics.

## Supplementary Material

ofag592_Supplementary_Data

## Supplementary Data

Supplementary materials are available at *Open Forum Infectious Diseases* online. Consisting of data provided by the authors to benefit the reader, the posted materials are not copyedited and are the sole responsibility of the authors, so questions or comments should be addressed to the corresponding author.
