# Supplementary material for: A Systematic Review and Network Meta-analysis of Empirical Antibiotics for Complicated Urinary Tract Infections
Source: Open Forum Infect Dis. 2026 Sep 26;13(10):ofag592. doi: 10.1093/ofid/ofag592 (PMC13642042; doi:10.1093/ofid/ofag592)
Supplement: Supplementary file 1 [file ofag592_supplementary_data.docx]

**Supplementary Materials**

*A Systematic Review and Network Meta-analysis of Empirical Antibiotics for Complicated Urinary Tract Infections*

**Table of Contents**

**Supplementary S1: Complete Search Strategies**

PubMed (MEDLINE)

EMBASE

Scopus

Cochrane CENTRAL

**Supplementary S2**

Risk of Bias Assessment

Certainty of Evidence — GRADE Assessment for Outcomes

**Supplementary S3: Subset Analyses**

All 47 Studies Included

Figure S1 — Network Plot: Clinical Response, All 47 Studies

Figure S2 — Forest Plot: Clinical Response, All 47 Studies

Only Studies After 2000

Figure S3 — Network Plot: Clinical Response, Studies After 2000

Figure S4 — Forest Plot: Clinical Response, Studies After 2000

Only Studies with Drugs Readily Available in the United States

Figure S5 — Network Plot: Clinical Response, US-Available Drugs

Figure S6 — Forest Plot: Clinical Response, US-Available Drugs

**Supplementary S4: Main Analysis**

Figure S7 — Network Plot: Microbiological Response, Studies After 2000 US-Available Drugs

# Supplementary S1: Complete Search Strategies

## PubMed (MEDLINE)

| **Search #** | **Search String** |
| --- | --- |
| #1 | "complicated urinary tract infection*"[tiab] OR "pyelonephritis"[tiab] OR ("hospital acquired"[tiab:~2] AND ("urinary tract infection"[tiab] OR "pyelonephritis"[tiab])) |
| #2 | Anti-Bacterial Agents[Mesh] OR "antibiotic*"[tiab] OR "antibacterial agent*"[tiab] OR "anti-bacterial agent*"[tiab] OR "anti-bacterial compound*"[tiab] OR "antibacterial compound*"[tiab] OR "anti-mycobacterial agent*"[tiab] OR "antimycobacterial agent*"[tiab] OR "bacteriocid*"[tiab] OR "cefepime, enmetazobactam drug combination" [nm] OR "cefepime-enmetazobactam"[tiab] OR "Cefiderocol"[Mesh] OR "cefiderocol*"[tiab] OR "S-649266"[tiab] OR "S649266"[tiab] OR "fetroja"[tiab] OR "Ceftaroline"[Mesh] OR "ceftaroline*"[tiab] OR "PPI-0903"[tiab] OR "PPI0903"[tiab] OR "TAK-599"[tiab] OR "TAK599"[tiab] OR "T-91825"[tiab] OR "T91825"[tiab] OR "teflaro"[tiab] OR "zinforo"[tiab] OR "avibactam, ceftazidime drug combination" [nm] OR "ceftazidime-avibactam"[tiab] OR "avycaz"[tiab] OR "ceftobiprole" [nm] OR "BAL9141"[tiab] OR "BAL 9141"[tiab] OR "zevtera"[tiab] OR "ceftolozane, tazobactam drug combination" [nm] OR "ceftolozane-tazobactam"[tiab] OR "ceftolozane sulfate tazobactam"[tiab] OR "zerbaxa"[tiab] OR "imipenem-cilastatin-relebactam"[tiab] OR "meropenem and vaborbactam" [nm] OR "meropenem-vaborbactam"[tiab] OR "meropenem vaborbactam"[tiab] OR "vabomere"[tiab] OR "sulbactam-durlobactam" [nm] OR "sulbactam-durlobactam"[tiab] OR "sulopenem" [nm] OR "CP 70429"[tiab] OR "CP-70,429"[tiab] OR "CP-70429"[tiab] OR "Cefepime"[Mesh] OR "cefepime*"[tiab] OR "cefepim"[tiab] OR "BMY 28142"[tiab] OR "BMY-28142"[tiab] OR "BMY28142"[tiab] OR "maxipime"[tiab] OR "cefepime hydrochloride"[tiab] OR "quadrocef"[tiab] OR "axépim"[tiab] OR "axepim"[tiab] OR "Piperacillin, Tazobactam Drug Combination"[Mesh] OR "piperacillin-tazobactam"[tiab] OR "piperacillin tazobactam"[tiab] OR "pipercillin sodium tazobactam sodium"[tiab] OR "zosyn"[tiab] OR "tazocillin"[tiab] OR "tazocel"[tiab] OR "tazocin"[tiab] OR "Meropenem"[Mesh] OR "meropenem"[tiab] OR "merrem"[tiab] OR "SM 7338"[tiab] OR "SM-7338"[tiab] OR "Ronem"[tiab] OR "penem"[tiab] OR "Vancomycin"[Mesh] OR "vancomycin*"[tiab] OR "vancocin"[tiab] OR "vancocine*"[tiab] OR "AB-vancomycin"[tiab] OR "vancomicina*"[tiab] OR "Linezolid"[Mesh] OR "linezolid*"[tiab] OR "zyvox"[tiab] OR "tedizolid"[nm] OR "tedizolid"[tiab] OR "torezolid"[tiab] OR "TR 700"[tiab] OR "TR700"[tiab] OR "telavancin"[nm] OR "telavancin*"[tiab] OR "TD 6424"[tiab] OR "TD6424"[tiab] OR "vibativ"[tiab] OR "quinupristin-dalfopristin"[nm] OR "quinupristin-dalfopristin"[tiab] OR "synercid"[tiab] OR "RP 59500"[tiab] OR "RP-59500"[tiab] OR "Ceftriaxone"[Mesh] OR "ceftriaxone"[tiab] OR "ceftriaxone-tobramycin"[tiab] OR "Rocephin"[tiab] OR "Imipenem"[Mesh] OR "imipenem"[tiab] OR "Cilastatin, Imipenem Drug Combination"[Mesh] OR "imipenem-cilastatin"[tiab] OR "Ertapenem"[Mesh] OR "ertapenem"[tiab] OR "TMP-SMX"[tiab] OR "bactrim"[tiab] OR "Trimethoprim, Sulfamethoxazole Drug Combination"[Mesh] OR "Trimethoprim-sulfamethoxazole" OR "cephalaxin"[tiab] OR "Cefazolin"[Mesh] OR "cefazolin"[tiab] OR "Ciprofloxacin"[Mesh] OR "ciproflaxacin"[tiab] OR "Levofloxacin"[Mesh] OR "levofloxacin"[tiab] OR "fluroquinolones"[tiab] OR "tebipenem pivoxil hydrobromide"[tiab] OR "plazomycin"[tiab] OR "finafloxacin"[nm] OR "finafloxacin"[tiab] OR "Doripenem"[Mesh] OR "doripenem"[tiab] OR "Amikacin"[Mesh] OR "amikacin"[tiab] OR "Gentamicins"[Mesh] OR "gentamicin"[tiab] OR "Tobramycin"[Mesh] OR "tobramycin"[tiab] OR "Cefotaxime"[Mesh] OR "cefotaxime"[tiab] |
| #3 | ("randomized controlled trial"[pt] OR "controlled clinical trial"[pt] OR "randomized"[tiab] OR "randomised"[tiab] OR "placebo"[tiab] OR "drug therapy"[sh] OR ("randomly"[tiab] OR "trial"[tiab] OR "groups"[tiab])) NOT ("animals"[MeSH Terms] NOT "humans"[MeSH Terms]) |
| #4 | #1 AND #2 AND #3 |
| #5 | ((infant[mesh] OR child[mesh] OR adolescent[mesh]) NOT adult[mesh]) |
| #6 | #4 NOT #5 |
| #7 | ("1990"[Date - Publication] : "3000"[Date - Publication]) |
| #8 | #6 AND #7 |

## EMBASE

| **Search #** | **Search String** |
| --- | --- |
| #1 | 'complicated urinary tract infection':ti,ab OR 'pyelonephritis'/exp OR 'pyelonephritis':ti,ab OR (((hospital NEAR/3 acquired):ti,ab) AND ('urinary tract infection':ti,ab OR 'pyelonephritis':ti,ab)) |
| #2 | 'antibiotic agent'/exp OR 'antibiotic*':ti,ab OR 'antibacterial agent*':ti,ab OR 'anti-bacterial agent*':ti,ab OR 'anti-bacterial compound*':ti,ab OR 'antibacterial compound*':ti,ab OR 'anti-mycobacterial agent*':ti,ab OR 'antimycobacterial agent*':ti,ab OR 'bacteriocid*':ti,ab OR 'cefepime plus enmetazobactam'/exp OR 'cefepime-enmetazobactam':ti,ab OR 'cefiderocol'/exp OR 'cefiderocol':ti,ab OR 's-649266':ti,ab OR 's649266':ti,ab OR 'fetroja':ti,ab OR 'ceftaroline'/exp OR 'ceftaroline':ti,ab OR 'ppi-0903':ti,ab OR 'ppi0903':ti,ab OR 'tak-599':ti,ab OR 'tak599':ti,ab OR 't-91825':ti,ab OR 't91825':ti,ab OR 'ceftaroline fosamil'/exp OR 'teflaro':ti,ab OR 'zinforo':ti,ab OR 'avibactam plus ceftazidime'/exp OR 'ceftazidime-avibactam':ti,ab OR 'avycaz':ti,ab OR 'ceftobiprole'/exp OR 'bal9141':ti,ab OR 'bal 9141':ti,ab OR 'ceftobiprole medocaril'/exp OR 'zevtera':ti,ab OR 'ceftolozane plus tazobactam'/exp OR 'ceftolozane-tazobactam':ti,ab OR 'ceftolozane sulfate tazobactam':ti,ab OR 'zerbaxa':ti,ab OR 'cilastatin sodium plus imipenem plus relebactam'/exp OR 'imipenem-cilastatin-relebactam':ti,ab OR 'meropenem plus vaborbactam'/exp OR 'meropenem-vaborbactam':ti,ab OR 'meropenem vaborbactam':ti,ab OR 'vabomere':ti,ab OR 'durlobactam plus sulbactam'/exp OR 'sulbactam-durlobactam':ti,ab OR 'sulopenem'/exp OR 'cp 70429':ti,ab OR 'cp-70,429':ti,ab OR 'cp-70429':ti,ab OR 'cefepime'/exp OR 'cefepime':ti,ab OR 'cefepim':ti,ab OR 'bmy 28142':ti,ab OR 'bmy-28142':ti,ab OR 'bmy28142':ti,ab OR 'maxipime':ti,ab OR 'cefepime hydrochloride':ti,ab OR 'quadrocef':ti,ab OR 'axepim':ti,ab OR 'piperacillin plus tazobactam'/exp OR 'piperacillin-tazobactam':ti,ab OR 'piperacillin tazobactam':ti,ab OR 'pipercillin sodium tazobactam sodium':ti,ab OR 'zosyn':ti,ab OR 'tazocillin':ti,ab OR 'tazocel':ti,ab OR 'tazocin':ti,ab OR 'meropenem'/exp OR 'meropenem':ti,ab OR 'merrem':ti,ab OR 'sm 7338':ti,ab OR 'sm-7338':ti,ab OR 'amoxicillin'/exp OR 'ronem':ti,ab OR 'penem':ti,ab OR 'vancomycin'/exp OR 'vancomycin*':ti,ab OR 'vancocin':ti,ab OR 'vancocine*':ti,ab OR 'ab-vancomycin':ti,ab OR 'vancomicina*':ti,ab OR 'linezolid'/exp OR 'linezolid*':ti,ab OR 'zyvox':ti,ab OR 'tedizolid'/exp OR 'tedizolid':ti,ab OR 'torezolid':ti,ab OR 'tr 700':ti,ab OR 'tr700':ti,ab OR 'telavancin'/exp OR 'telavancin*':ti,ab OR 'td 6424':ti,ab OR 'td6424':ti,ab OR 'vibativ':ti,ab OR 'dalfopristin plus quinupristin'/exp OR 'quinupristin-dalfopristin':ti,ab OR 'synercid':ti,ab OR 'rp 59500':ti,ab OR 'rp-59500':ti,ab OR 'ceftriaxone'/exp OR 'ceftriaxone':ti,ab OR 'ceftriaxone-tobramycin':ti,ab OR 'rocephin':ti,ab OR 'imipenem'/exp OR 'cilastatin plus imipenem'/exp OR 'imipenem':ti,ab OR 'imipenem-cilastatin':ti,ab OR 'ertapenem'/exp OR 'ertapenem':ti,ab OR 'tmp-smx':ti,ab OR 'cotrimoxazole'/exp OR 'bactrim':ti,ab OR 'trimethoprim-sulfamethoxazole':ti,ab OR 'cephalaxin':ti,ab OR 'cefazolin'/exp OR 'cefazolin':ti,ab OR 'ciprofloxacin'/exp OR 'ciproflaxacin':ti,ab OR 'levofloxacin'/exp OR 'levofloxacin':ti,ab OR 'fluroquinolones':ti,ab OR 'tebipenem pivoxil'/exp OR 'tebipenem pivoxil hydrobromide':ti,ab OR 'plazomycin':ti,ab OR 'finafloxacin'/exp OR 'finafloxacin':ti,ab OR 'doripenem'/exp OR 'doripenem':ti,ab OR 'amikacin'/exp OR 'amikacin':ti,ab OR 'gentamicin'/exp OR 'gentamicin':ti,ab OR 'tobramycin'/exp OR 'tobramycin':ti,ab OR 'cefotaxime'/exp OR 'cefotaxime':ti,ab |
| #3 | ('randomized controlled trial'/exp OR 'controlled clinical trial'/de OR random*:ti,ab,tt OR 'randomization'/de OR 'intermethod comparison'/de OR placebo:ti,ab,tt OR compare:ti,tt OR compared:ti,tt OR comparison:ti,tt OR ((evaluated:ab OR evaluate:ab OR evaluating:ab OR assessed:ab OR assess:ab) AND (compare:ab OR compared:ab OR comparing:ab OR comparison:ab)) OR ((open NEXT/1 label):ti,ab,tt) OR (((double OR single OR doubly OR singly) NEXT/1 (blind OR blinded OR blindly)):ti,ab,tt) OR 'double blind procedure'/de OR ((parallel NEXT/1 group*):ti,ab,tt) OR crossover:ti,ab,tt OR 'cross over':ti,ab,tt OR (((assign* OR match OR matched OR allocation) NEAR/6 (alternate OR group OR groups OR intervention OR interventions OR patient OR patients OR subject OR subjects OR participant OR participants)):ti,ab,tt) OR assigned:ti,ab,tt OR allocated:ti,ab,tt OR ((controlled NEAR/8 (study OR design OR trial)):ti,ab,tt) OR volunteer:ti,ab,tt OR volunteers:ti,ab,tt OR 'human experiment'/de OR trial:ti,tt) NOT (((random* NEXT/1 sampl* NEAR/8 ('cross section*' OR questionnaire* OR survey OR surveys OR database OR databases)):ti,ab,tt) NOT ('comparative study'/de OR 'controlled study'/de OR 'randomised controlled':ti,ab,tt OR 'randomized controlled':ti,ab,tt OR 'randomly assigned':ti,ab,tt) OR ('cross-sectional study' NOT ('randomized controlled trial'/exp OR 'controlled clinical trial'/de OR 'controlled study'/de OR 'randomised controlled':ti,ab,tt OR 'randomized controlled':ti,ab,tt OR 'control group':ti,ab,tt OR 'control groups':ti,ab,tt)) OR ('case control*':ti,ab,tt AND random*:ti,ab,tt NOT ('randomised controlled':ti,ab,tt OR 'randomized controlled':ti,ab,tt)) OR ('systematic review':ti,tt NOT (trial:ti,tt OR study:ti,tt)) OR (nonrandom*:ti,ab,tt NOT random*:ti,ab,tt) OR 'random field*':ti,ab,tt OR (('random cluster' NEAR/4 sampl*):ti,ab,tt) OR (review:ab AND review:it NOT trial:ti,tt) OR ('we searched':ab AND (review:ti,tt OR review:it)) OR 'update review':ab OR ((databases NEAR/5 searched):ab) OR ((rat:ti,tt OR rats:ti,tt OR mouse:ti,tt OR mice:ti,tt OR swine:ti,tt OR porcine:ti,tt OR murine:ti,tt OR sheep:ti,tt OR lambs:ti,tt OR pigs:ti,tt OR piglets:ti,tt OR rabbit:ti,tt OR rabbits:ti,tt OR cat:ti,tt OR cats:ti,tt OR dog:ti,tt OR dogs:ti,tt OR cattle:ti,tt OR bovine:ti,tt OR monkey:ti,tt OR monkeys:ti,tt OR trout:ti,tt OR marmoset*:ti,tt) AND 'animal experiment'/de) OR ('animal experiment'/de NOT ('human experiment'/de OR 'human'/de))) |
| #4 | #1 AND #2 AND #3 |
| #5 | ([infant]/lim OR [child]/lim OR [adolescent]/lim) NOT [adult]/lim |
| #6 | #4 NOT #5 |
| #7 | #6 AND [1990-2025]/py |

## Scopus

| **Search #** | **Search String** |
| --- | --- |
| #1 | TITLE-ABS-KEY({complicated urinary tract infection} OR pyelonephritis OR ((hospital W/2 acquired) AND ({urinary tract infection} OR pyelonephritis))) |
| #2 | TITLE-ABS-KEY(antibiotic* OR {antibacterial agent*} OR {anti-bacterial agent*} OR {anti-bacterial compound*} OR {antibacterial compound*} OR {anti-mycobacterial agent*} OR {antimycobacterial agent*} OR bacteriocid* OR {cefepime-enmetazobactam} OR cefiderocol OR {S-649266} OR S649266 OR fetroja OR ceftaroline OR {PPI-0903} OR PPI0903 OR {TAK-599} OR TAK599 OR {T-91825} OR T91825 OR teflaro OR zinforo OR {ceftazidime-avibactam} OR avycaz OR BAL9141 OR {BAL 9141} OR zevtera OR {ceftolozane-tazobactam} OR {ceftolozane sulfate tazobactam} OR zerbaxa OR {imipenem-cilastatin-relebactam} OR {meropenem-vaborbactam} OR {meropenem vaborbactam} OR vabomere OR {sulbactam-durlobactam} OR {CP 70429} OR {CP-70,429} OR {CP-70429} OR cefepime OR cefepim OR {BMY 28142} OR {BMY-28142} OR BMY28142 OR maxipime OR {cefepime hydrochloride} OR quadrocef OR axepim OR {piperacillin-tazobactam} OR {piperacillin tazobactam} OR {pipercillin sodium tazobactam sodium} OR zosyn OR tazocillin OR tazocel OR tazocin OR meropenem OR merrem OR {SM 7338} OR {SM-7338} OR Ronem OR penem OR vancomycin OR vancocin OR vancocine OR {AB-vancomycin} OR vancomicina OR linezolid OR zyvox OR tedizolid OR torezolid OR {TR 700} OR TR700 OR telavancin OR {TD 6424} OR TD6424 OR vibativ OR {quinupristin-dalfopristin} OR synercid OR {RP 59500} OR {RP-59500} OR ceftriaxone OR {ceftriaxone-tobramycin} OR rocephin OR imipenem OR {imipenem-cilastatin} OR ertapenem OR {TMP-SMX} OR bactrim OR {Trimethoprim-sulfamethoxazole} OR cephalaxin OR cefazolin OR ciproflaxacin OR levofloxacin OR fluroquinolones OR {tebipenem pivoxil hydrobromide} OR plazomycin OR finafloxacin OR doripenem OR amikacin OR gentamicin OR tobramycin OR cefotaxime) |
| #3 | TITLE-ABS-KEY ( {Clinical-trial} OR {controlled-trial} OR randomi* OR randomly OR ( random W/4 ( allocat* OR distribut* OR assign* ) ) OR {placebo} OR {trial} OR {groups} OR {subgroups} ) OR TITLE ( rct ) |
| #4 | #1 AND #2 AND #3 |
| #5 | #4 AND [1990-2025]/py NOTE: There is not a validated search hedge for age in Scopus, nor is there an appropriate filter. Studies with children had to be screened out manually. |

## Cochrane CENTRAL

| **Search #** | **Search String** |
| --- | --- |
| #1 | ("complicated urinary tract infection" OR pyelonephritis OR ((hospital NEAR/2 acquired) AND ("urinary tract infection" or pyelonephritis))):ti,ab,kw |
| #2 | [mh "anti-bacterial agents"] OR [mh "cefderocol"] OR [mh "ceftaroline"] OR [mh "cefepime"] OR [mh "piperacillin, tazobactam drug combination"] OR [mh "meropenem"] OR [mh "vancomycin"] OR [mh "linezolid"] OR [mh "ceftriaxone"] OR [mh "imipenem"] OR [mh "cilastatin, imipenem drug combination"] OR [mh "ertapenem"] OR [mh "trimethoprim, sulfamethoxazole drug combination"] OR [mh "cefazolin"] OR [mh "ciprofloxacin"] OR [mh "levofloxacin"] OR [mh "doripenem"] OR [mh "amikacin"] OR [mh "gentamycins"] OR [mh "tobramycin"] OR [mh cefotaxime] OR (antibiotic OR "antibacterial agents" OR "anti-bacterial agents" OR "anti-bacterial compound" OR "antibacterial compounds" OR "anti-mycobacterial agent" OR "antimycobacterial agents" OR bacteriocide OR "cefepime-enmetazobactam" OR cefiderocol OR "S-649266" OR s649266 OR fetroja OR ceftaroline OR "PPI-0903" OR ppi0903 OR "TAK-599" OR tak599 OR "T-91825" OR t91825 OR teflaro OR zinforo OR "ceftazidime-avibactam" OR avycaz OR bal9141 OR "BAL 9141" OR zevtera OR "ceftolozane-tazobactam" OR "ceftolozane sulfate tazobactam" OR zerbaxa OR "imipenem-cilastatin-relebactam" OR "meropenem-vaborbactam" OR "meropenem vaborbactam" OR vabomere OR "sulbactam-durlobactam" OR "CP 70429" OR "CP-70,429" OR "CP-70429" OR cefepime OR cefepim OR "BMY 28142" OR "BMY-28142" OR bmy28142 OR maxipime OR "cefepime hydrochloride" OR quadrocef OR axepim OR "piperacillin-tazobactam" OR "piperacillin tazobactam" OR "pipercillin sodium tazobactam sodium" OR zosyn OR tazocillin OR tazocel OR tazocin OR meropenem OR merrem OR "SM 7338" OR "SM-7338" OR ronem OR penem OR vancomycin OR vancocin OR vancocine OR "AB-vancomycin" OR vancomicina OR linezolid OR zyvox OR tedizolid OR torezolid OR "TR 700" OR tr700 OR telavancin OR "TD 6424" OR td6424 OR vibativ OR "quinupristin-dalfopristin" OR synercid OR "RP 59500" OR "RP-59500" OR ceftriaxone OR "ceftriaxone-tobramycin" OR rocephin OR imipenem OR "imipenem-cilastatin" OR ertapenem OR "TMP-SMX" OR bactrim OR "Trimethoprim-sulfamethoxazole" OR cephalaxin OR cefazolin OR ciproflaxacin OR levofloxacin OR fluroquinolones OR "tebipenem pivoxil hydrobromide" OR plazomycin OR finafloxacin OR doripenem OR amikacin OR gentamicin OR tobramycin OR cefotaxime):ti,ab,kw |
| #3 | #1 AND #2 |
| #4 | Limited 1990–2025 |

# Supplementary S2

## Risk of Bias Assessment

| **Study (Author, Year)** | **D1** | **D2** | **D3** | **D4** | **D5** | **Overall RoB** |
| --- | --- | --- | --- | --- | --- | --- |
| Dunne 2023 | 🟢 | 🟢 | 🟢 | 🟢 | 🟢 | 🟢 Low |
| Eckburg 2022 | 🟢 | 🟢 | 🟢 | 🟢 | 🟢 | 🟢 Low |
| Tehrani 2021 | 🔴 | 🔴 | 🟢 | 🟡 | 🟢 | 🔴 High |
| Kaye 2019 | 🟢 | 🟢 | 🟢 | 🟢 | 🟢 | 🟢 Low |
| Kaye 2018 | 🟢 | 🟢 | 🟢 | 🟢 | 🟢 | 🟢 Low |
| Portsmouth 2018 | 🟢 | 🟢 | 🟢 | 🟢 | 🟢 | 🟢 Low |
| Sims 2017 | 🟢 | 🟢 | 🟢 | 🟢 | 🟢 | 🟢 Low |
| Wagenlehner 2016 | 🟢 | 🟢 | 🟢 | 🟢 | 🟢 | 🟢 Low |
| Wagenlehner 2015 | 🟢 | 🟢 | 🟢 | 🟢 | 🟢 | 🟢 Low |
| Vasquez 2012 | 🟢 | 🟢 | 🟢 | 🟢 | 🟢 | 🟢 Low |
| Park 2012 | 🟢 | 🟢 | 🟡 | 🟢 | 🟢 | 🟡 Concerns |
| Ebrahimzadeh 2010 | 🟡 | 🔴 | 🟢 | 🟡 | 🟢 | 🔴 High |
| Naber 2009 | 🔴 | 🟢 | 🟢 | 🟢 | 🟢 | 🔴 High |
| Peterson 2008 | 🟢 | 🟡 | 🟢 | 🟢 | 🟢 | 🟡 Concerns |
| Tomera 2002 | 🟢 | 🟢 | 🟢 | 🟢 | 🟢 | 🟢 Low |
| Naber 2002 | 🟢 | 🟢 | 🟡 | 🟢 | 🟢 | 🟡 Concerns |
| Jimenez-Cruz 2002 | 🟡 | 🟢 | 🟢 | 🟢 | 🟢 | 🟡 Concerns |

Risk of bias was assessed using the Cochrane Risk of Bias 2 (RoB 2) tool. Each study was evaluated across five domains: D1 – Bias arising from the randomization process; D2 – Bias due to deviations from intended interventions; D3 – Bias due to missing outcome data; D4 – Bias in measurement of the outcome; D5 – Bias in selection of the reported result. Each domain was rated as low risk (🟢), some concerns (🟡), or high risk (🔴). The overall risk of bias judgment was determined by the highest-risk domain rating.

## Certainty of Evidence — GRADE Assessment for Outcomes

| **Study (Author, Year)** | **Certainty of Evidence: Clinical Cure‡** | **Certainty of Evidence: Microbiological Cure‡** |
| --- | --- | --- |
| Dunne 2023 | 🟢 High | 🟢 High |
| Eckburg 2022 | 🟢 High | 🟢 High |
| Tehrani 2021 | 🔴 Very Low | 🔴 Very Low |
| Kaye 2019 (ZEUS) | 🟢 High | 🟡 Moderate |
| Kaye 2018 (TANGO) | 🟢 High | 🟢 High |
| Portsmouth 2018 | 🟢 High | 🟡 Moderate |
| Sims 2017 | 🟡 Moderate | 🟡 Moderate |
| Wagenlehner 2016 | 🟢 High | 🟢 High |
| Wagenlehner 2015 | 🟢 High | 🟢 High |
| Vazquez 2012 | 🟡 Moderate | 🔴 Low |
| Park 2012 | 🟡 Moderate | 🟡 Moderate |
| Ebrahimzadeh 2010 | 🔴 Very Low | 🔴 Very Low |
| Naber 2009 | 🟡 Moderate | 🟡 Moderate |
| Peterson 2008 | 🟡 Moderate | 🟡 Moderate |
| Jimenez-Cruz 2002 | 🟡 Moderate | 🟡 Moderate |
| Naber 2002 | 🟡 Moderate | 🟡 Moderate |
| Tomera 2002 | 🟡 Moderate | 🟡 Moderate |

Certainty of evidence was assessed using the GRADE (Grading of Recommendations Assessment, Development, and Evaluation) framework adapted for network meta-analysis. Each study contribution was evaluated across five domains: risk of bias, imprecision, inconsistency, indirectness, and publication bias. Evidence was rated as high (🟢), moderate (🟡), low, or very low (🔴). Downgrading occurred when serious or very serious concerns were identified in one or more domains. The symbol ‡ denotes that ratings reflect the certainty of evidence for the individual trial's contribution to the network, not the overall network estimate.

# Supplementary S3: Subset Analyses

## All 47 Studies Included

### Figure S1. Network Plot: Clinical Response, All 47 Studies

*Figure S1. Circular network plot for clinical response — all 47 included studies. Node size is proportional to the total number of patients per treatment arm; edge thickness reflects the number of direct comparisons between nodes.*

### Figure S2. Forest Plot: Clinical Response, All 47 Studies

*Figure S2. Forest plot for clinical response all 47 included studies. Odds ratios (ORs) and 95% confidence intervals (CIs) are shown relative to ceftriaxone (OR = 1.00). Values >1 favor the comparator; values <1 favor ceftriaxone.*

## Studies Published After 2000

### Figure S3. Network Plot: Clinical Response, Studies After 2000

*Figure S3. Circular network plot for clinical response — studies published from 2000 onward. Node size is proportional to the total number of patients per treatment arm; edge thickness reflects the number of direct comparisons between nodes. Ceftriaxone serves as the reference comparator.*

### Figure S4 - Forest Plot: Clinical Response, Studies Published After year 2000

*Figure S4. Forest plot for clinical response. Studies published from 2000 onward. ORs and 95% CIs relative to ceftriaxone (OR = 1.00). Values >1 favor the ceftriaxone; values <1 favor compartor.*

## Only Studies with Drugs Readily Available in the United States

### Figure S5 — Network Plot: Clinical Response, US-Available Drugs

*Figure S5. Circular network plot for clinical response — studies restricted to drugs currently available in the United States. Node size is proportional to the total number of patients per treatment arm; edge thickness reflects the number of direct comparisons between nodes.*

### Figure S6 - Forest Plot: Clinical Response, US-Available Drugs

*Figure S6. Forest plot for clinical response. Studies restricted to drugs currently available in the United States. ORs and 95% CIs relative to ceftriaxone (OR = 1.00). Values >1 favor ceftriaxone; values <1 favor compartor.*

# Supplementary S4: Main Analysis

### Figure S7 - Network Plot: Microbiological Response, Studies After 2000 for US-Available Drugs

*Figure S7. Circular network plot for microbiological response — studies published from 2000 onward to drugs currently available in the United States. Node size is proportional to the total number of patients per treatment arm; edge thickness reflects the number of direct comparisons between nodes*
